# Interferon-Gamma and Interlukin-4 Patterns in BALB/c Mice Suffering From Cutaneous Leishmaniasis Treated With Cantharidin

**DOI:** 10.5812/jjm.10907

**Published:** 2014-06-01

**Authors:** Yahya Maroufi, Fatemeh Ghaffarifar, Abdolhosein Dalimi, Zohreh Sharifi

**Affiliations:** 1 Department of Parasitology, Faculty of Medical Sciences, Zabol University of Medical Sciences, Zabol, IR Iran; 2 Department of Parasitology, Faculty of Medical Sciences, Tarbiat Modares University, Tehran, IR Iran; 3 Department of Virology, Iranian Blood Transfusion organization, Tehran, IR Iran

**Keywords:** Mice, Inbred BALB/c, Cantharidin, Leishmaniasis, Cutaneous, Interferon-gamma

## Abstract

**Background:**

Cutaneous leishmaniasis is a health problem in the world. Lesions should be treated on cosmetically or functionally important sites, such as the face and hands. Cantharidin is a terpenoid compound produced naturally by beetles of Meloidae and Oedemeridae families.

**Objectives:**

The current study aimed to investigate the effect of cantharidin on Cutaneous Leishmaniasis (CL) lesions and IFN-γ and IL-4 patterns in infected BALB/c mice.

**Materials and Methods:**

Infected BALB/c mice were divided into five groups as: untreated (control group), eucerin-treated and 0.05%, 0.1% and 0.5% cantharidin-treated. Lesions diameter was measured by Vernier caliper every three days for four weeks. Cytokines levels were measured by enzyme-linked immunosorbent assay (ELISA) using U-CyTech kit.

**Results:**

The results indicated that treatment with cantharidin exacerbates lesions compared with the controls, except for 0.05% cantharidin dose that restrained lesion growth significantly. Interferon gamma level in cantharidin-treated groups was significantly less than that of the control group. But interlukin-4 level was similar among the groups.

**Conclusions:**

The current study results indicated that high doses of cantharidin exacerbates leishmaniasis lesion, but low dose of cantharidin inhibits lesion growth.

## 1. Background

Cutaneous leishmaniasis is one of the health problems in the world. About 12 million people are infected by *Leishmania* in 88 countries around world (1.5 to 2 million new cases each year). Cutaneous Leishmaniasis (CL) is caused by variable species of *Leishmania *such as *Leishmania major*. Clinical manifestation of CL is characterized by ulcerative skin lesions developing at the site of sandfly bite. Lesions should be treated on cosmetically or functionally important sites, such as the face and hands. The pentavalent antimony sodium stibogluconate and meglumine antimoniate are the main chemotherapy. In addition to side effects, resistance and relapse happen (1, 2). Cantharidin is a terpenoid compound produced naturally by families of Meloidae and Oedemeridae beetles (3). Chinese have used it as a traditional medicine about 2000 years ago (4). It has been used to treat wart and cutaneous lesions (4, 5). Hakim Jorjani used cantharidin to treat wart, hair loss, rabidity and black nails (6).

Cantharidin is a protein phosphatase 1 and 2A (PP1 & 2A) inhibitor, and PP1 and 2A are primary targets of cantharidin (3,7, 8). There are some studies about the effect of cantharidin on several cancer cells (9-12). Cantharidin induces apoptosis in cancer cells and also in *L. major* in vitro and in vivo (13-15). In experimental leishmaniasis, immunity is principally mediated by T lymphocytes. T helper (Th1)and Th2 cells can be identified by the cytokines they secrete: Th1 cells secrete activators of cell-mediated immunity such as IFN-γ, while Th2 cells secrete cytokines such as IL-4, which promote antibody responses (16). Interferon-gamma (IFN-ϒ) is the essential cytokine for inducing protective immunity against cutaneous leishmaniasis. IFN-ϒ kills the parasite and causes protective immunity in both human and murine cutaneous leishmaniasis (16, 17). But, BALB/c mice that develop a typical Th2 response are highly susceptible to leishmaniasis (18).

## 2. Objectives

The current study aimed to investigate the effect of cantharidin on CL lesions and Interferon-gamma (IFN-ϒ) (as an indicator of Th1-type response), and Interlukin-4 (IL-4) (as a Th2-type response indicator) patterns in BALB/c mice infected with *L. major*.

## 3. Materials and Methods

### 3.1. Animals

Six eight-week-old female BALB/c mice were purchased from Razi Institute (Tehran, Iran).They were fed standard mouse chow and ad libitum water.

### 3.2. Parasites

*L. major* MRHO/IR/75/ER was used in this study, the parasite was maintained by passage through BALB/c mice and culture in NNN medium.

### 3.3. Mice Infecting

Thirty female BALB/c mice were infected by injecting 2×10^6^/mL stationary phase *L. major* promastigotes into the base of tail. The mice were divided into five groups as: untreated (control), eucerin-treated, 0.05%, 0.1% and 0.5% cantharidin-treated groups.

### 3.4. Cantharidin Preparation

Cantharidin was purchased from Sigma (Germany). It was dissolved in eucerin as ointment base. Cantharidin ointment was prepared in three doses (0.05%, 0.1% and 0.5%). Cantharidin was used once a day for four weeks topically.

### 3.5. Lesion Size and Cytokines Level Measurement

Cutaneous leishmaniasis lesions diameter was measured by Vernier caliper every three days for four weeks. To measure IFN-ϒ and IL-4 level, mice were killed at the end of treatment. Spleen lymphocytes were extracted and 1 x 10^6^/mL lymphocytes was cultured in 24-well plates in the RPMI-1640 cell culture medium (GibCo, USA) containing 10% heat-inactivated fetal calf serum (FCS; GibCo, USA) and 100 U/mL penicillin, 100 μg/mL streptomycin (Sigma, Germany). Soluble *Leishmania* antigens (SLA) were obtained by resuspending *L. major* promastigotes in sterile PBS at a concentration about 108 parasite/mL. Parasites were lysed by five freeze-thawing cycles, and then centrifuged at 4°C for 15 minutes. The supernatant was collected and protein concentration was measured by Bradford assay. Soluble *Leishmania* antigens, by concentration of 20 μg/mL, were added to wells and then plates incubated in 5% CO_2_ at 37°C. Supernatants were collected after 48 and 72 hours and stored at -80°C until use. Cytokines levels were measured by enzyme-linked immunosorbent assay (ELISA) using U-CyTech (bioscience, Netherlands) kit according to the manufacturer’s instructions.

### 3.6. Statistical Analysis

Data were analyzed using a one-way analysis of variance (ANOVA). Results were shown as mean ± standard deviation (SD). All statistical analyses were performed using SPSS 16 software for Windows.

## 4. Results

### 4.1. Lesion Size

Table 1 shows the lesion size mean before and after treatment in different groups. The results of the current study indicated that treatment with cantharidin exacerbates lesions compared with the controls, excluding 0.05% dose. But 0.05% cantharidin restrained lesion growth significantly (P < 0.05).

**Table1.** Lesion Size in Different Groups Before and After Treatment^a^

Groups	Before Treatment	After Treatment
**Untreated (control) **	2.50 ± 0.50	3.70 ± 0.18
**Eucerin-treated**	5.66 ± 1.33	7.19 ± 0.59
**Cantharidin-treated**		
0.05%	4.6 ± 0.60	4.54 ± 0.19
0.1%	5.7 ± 1.00	6.13 ± 0.33
0.5%	7.33 ± 0.44	10.28 ± 0.29

^a^ Data showed as Mean ± SD.

### 4.2. IFN-ϒ and IL-4 Pattern

**Figure 1.**
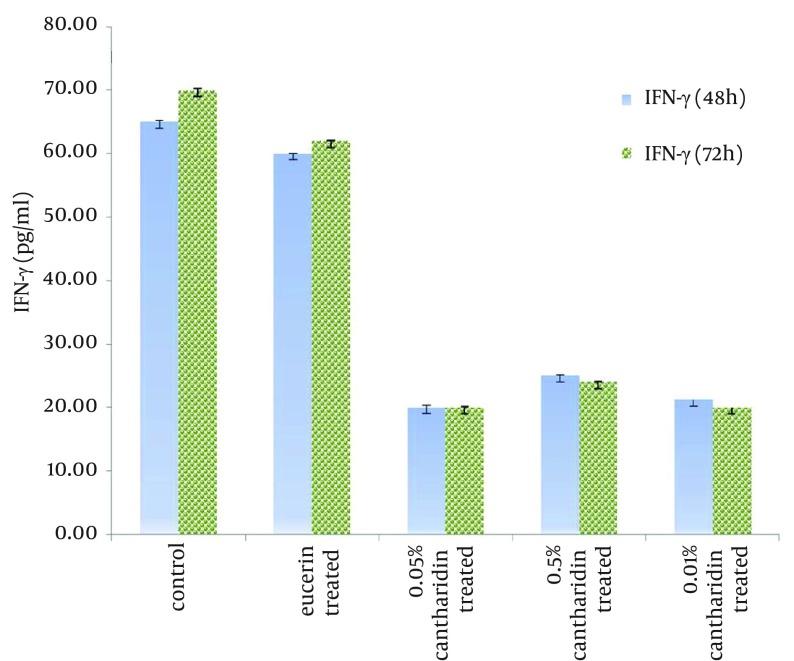
IFN-ϒ Level in Lymphocytes Separated From Spleen in different Groups After 48 and 72 Hours Following Treated With Soluble *Leishmania* Antigen

**Figure 2.**
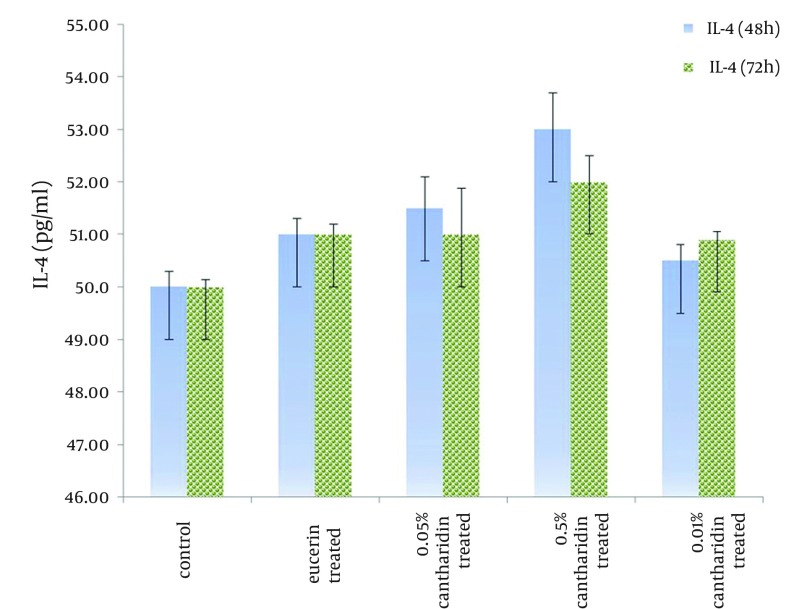
IL-4 Level in Lymphocytes Separated From Spleen in Different Groups After 48 and 72 Hous Following Treated With Soluble *Leishmania *Antigen

Control group and eucerin-treated group produced high levels of IFN-ϒ, but cantharidin-treated groups showed low levels of IFN-ϒ, significantly (P < 0.05) (Figure 1). There was no significant difference between IFN-ϒ levels in eucerin-treated and control groups. Interlukin-4 level was similar among the groups (Figure 2).

## 5. Discussion

This is the first report showing the effect of cantharidin on IFN-ϒ and IL-4 in cutaneous leishmaniasis. Results showed that cantharidin exacerbates CL lesion, except for 0.05% cantharidin. Cantharidin suppresses Th1 type response by inhibiting IFN-ϒ production, but it has no effect on IL-4. In the current study, treatment with 0.05% cantharidin restrained lesion growth, but it was unable to increase IFN-ϒ production. Some studies indicate the inflammatory reaction in blister location with lymphocytes and macrophages infiltration. Neutrophils are primary antimicrobial effector cells, and their main function is affecting phagocytosis and destroying invading pathogens. Following *L. major* transmission, neutrophils were observed capturing parasites rapidly and efficiently. Neutrophils produce and secret myeloperoxidase causing tissue damage (19-21). Cantharidin induces neutrophils infiltration into the blister site in the first 24 hours and macrophages from 24 to 72 hours (19, 21). Infiltrating neutrophils did not destroy the parasite. Dendritic cells produce IL-12, which drives the generation of Th1 cells. Th1 cells in turn activate macrophages to increase inducible nitric oxide synthases (iNOS) and nitric oxide (NO) production, which results in killing the intracellular parasites (20). Cantharidin arrests dendritic cells proliferation and IFN-ϒ production (22). Norcantharidin, demethylated form of cantharidin, inhibits peripheral blood mononuclear cells (PBMC) proliferation in vitro (23). Also it can inhibit IL-12 production in human leukemic Jurket T cells, but doses less than 2 μg/mL induce IL-12 production (24).

The current study results indicate that high doses of cantharidin exacerbates lesion due to Th1-type response, therefore inhibiting IFN-ϒ production. But lesion growth in the group treated with 0.05% cantharidin was restrained. It can be supposed that cantharidin with low dose accelerates lesion healing.
